# Conservation of the C-type lectin fold for accommodating massive sequence variation in archaeal diversity-generating retroelements

**DOI:** 10.1186/s12900-016-0064-6

**Published:** 2016-08-31

**Authors:** Sumit Handa, Blair G. Paul, Jeffery F. Miller, David L. Valentine, Partho Ghosh

**Affiliations:** 1 Department of Chemistry & Biochemistry, University of California, San Diego, La Jolla, CA 92093 USA; 2 Marine Science Institute, University of California, Santa Barbara, CA 93106 USA; 3 Departments of Microbiology, Immunology, and Molecular Genetics, Molecular Biology Institute, and California NanoSystems Institute, University of California, Los Angeles, CA 90095 USA; 4 Department of Earth Science, University of California, Santa Barbara, CA 93106 USA

## Abstract

**Background:**

Diversity-generating retroelements (DGRs) provide organisms with a unique means for adaptation to a dynamic environment through massive protein sequence variation. The potential scope of this variation exceeds that of the vertebrate adaptive immune system. DGRs were known to exist only in viruses and bacteria until their recent discovery in archaea belonging to the ‘microbial dark matter’, specifically in organisms closely related to *Nanoarchaeota*. However, *Nanoarchaeota* DGR variable proteins were unassignable to known protein folds and apparently unrelated to characterized DGR variable proteins.

**Results:**

To address the issue of how *Nanoarchaeota* DGR variable proteins accommodate massive sequence variation, we determined the 2.52 Å resolution limit crystal structure of one such protein, AvpA, which revealed a C-type lectin (CLec)-fold that organizes a putative ligand-binding site that is capable of accommodating 10^13^ sequences. This fold is surprisingly reminiscent of the CLec-folds of viral and bacterial DGR variable protein, but differs sufficiently to define a new CLec-fold subclass, which is consistent with early divergence between bacterial and archaeal DGRs. The structure also enabled identification of a group of AvpA-like proteins in multiple putative DGRs from uncultivated archaea. These variable proteins may aid *Nanoarchaeota* and these uncultivated archaea in symbiotic relationships.

**Conclusions:**

Our results have uncovered the widespread conservation of the CLec-fold in viruses, bacteria, and archaea for accommodating massive sequence variation. In addition, to our knowledge, this is the first report of an archaeal CLec-fold protein.

## Background

Diversity-generating retroelements (DGRs) create massive sequence variation (10^12-20^) in select proteins. The only parallel for this scale of variation occurs in the vertebrate immune system [1]. Massive sequence variation enables adaptation to a dynamic environment, as seen for the prototypical *Bordetella* bacteriophage DGR [2], just as it does in the vertebrate immune system. DGRs have been identified in ecologically diverse bacteria, including members of the human microbiome, and in numerous viruses of bacteria [3–7]. Recently, DGRs were also identified in the third domain of life, archaea, from single-cell sequencing data of organisms that were uncultivated and harvested from a subterranean environment [8]. These organisms are related to *Nanoarchaeota*, which are nanosized, hyperthermophilic organisms that exist in symbiotic relationship with larger archaea [9, 10]. Although the single-cell sequenced organisms were not directly visualized, their genomic sequences support the hypothesis that these archaeal DGRs belong to nanosized, symbiotic organisms. Along with the archaeal DGRs, a putative virus of methanotrophic archaea, ANMV-1, was also identified to encode a DGR [8].

**Fig. 1:**
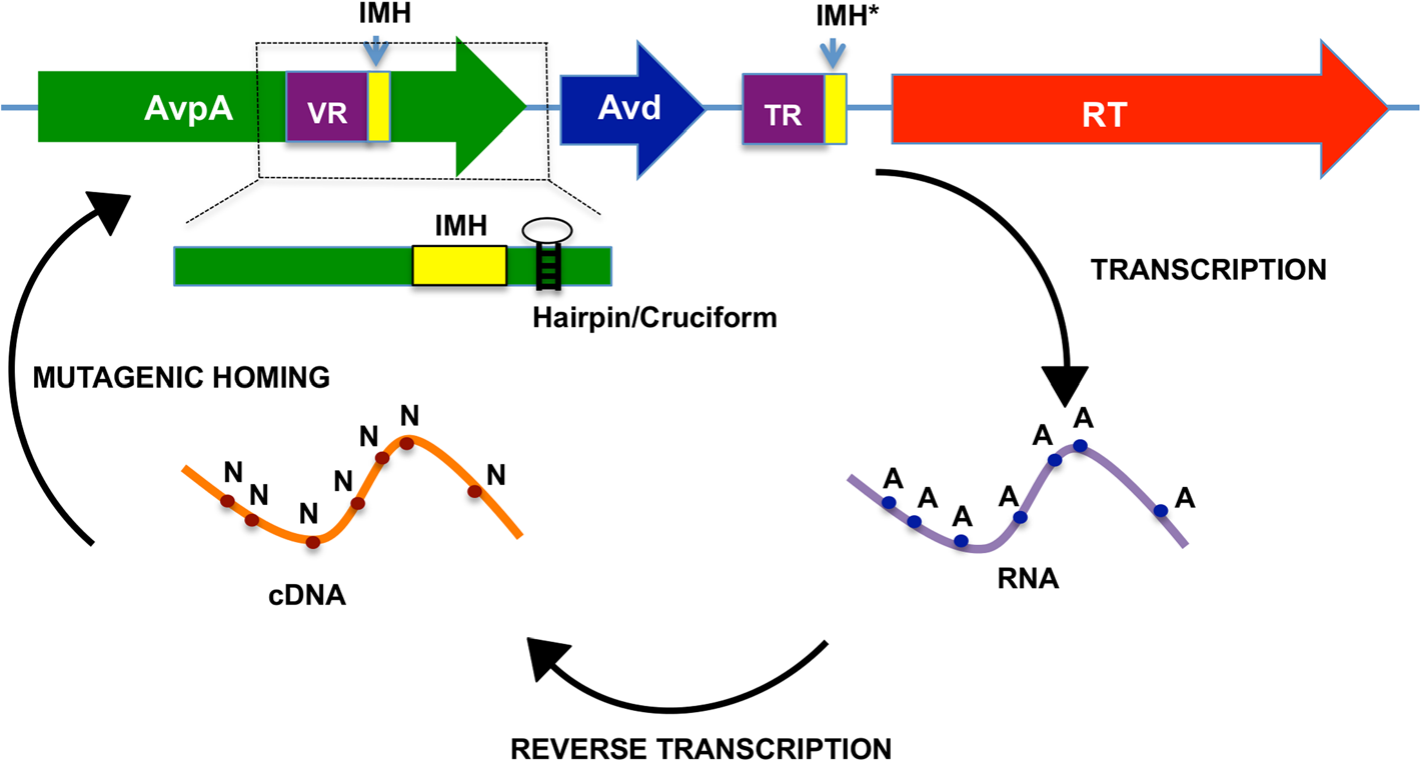
Schematic of DGR. Genetic information is transferred from an invariant TR to the VR of a variable protein (in this case AvpA), requiring the action of a reverse transcriptase (RT) and an accessory variability determinant (Avd) protein. This process involves reverse transcription of an RNA encoding the TR, and is accompanied by adenine-specific mutagenesis of the TR sequence. The mutagenized cDNA homes to the variable protein locus, and replaces the sequence information in the VR, resulting in a variant of the variable protein

The archaeal DGRs have in common the genetic elements identified in bacterial DGRs (Fig. 1). This includes a variable region (VR) that is located within the coding region of a variable protein, a template region (TR) that is similar (~90 % typically) but not identical to the VR and located in a proximal noncoding region, and a reverse transcriptase (RT) [3]. Genetic information is transferred from the TR to the VR through an RNA intermediate, a process termed retrohoming. In DGRs, retrohoming is accompanied by adenine-specific mutagenesis of sequence information. Thus, a hallmark of DGRs is the substitution of adenines in the TR by other bases in the VR, resulting in protein coding variation. Archaeal elements display this hallmark pattern of adenine substitution. Along with these core DGR components, the archaeal DGRs contain initiation of mutagenic homing sequences in the VR (i.e., IMH) and TR (i.e., IMH*) (Fig. 1). These elements differ slightly in sequence, and have been documented in the *Bordetella* bacteriophage (Bb) DGR to specify the directionality of information transfer [3]. That is, the region containing the IMH* (i.e., TR) serves as the invariant source of sequence information, and the region containing the IMH (i.e., VR) serves as the recipient of that (mutagenized) sequence information. In addition, a hairpin/cruciform structure downstream of the VR in evident in the archaeal DGR, and in the Bb DGR this element was seen to increase the efficiency of homing [11]. Proteins were also identified with similar physical properties to the accessory variability determinant (Avd), which in the Bb DGR binds RT and is required for retrohoming [12].

The variable protein encoded by ANMV-1 was classifiable by sequence using Phyre [8, 13]. This variable protein was predicted to be structurally similar to the Bb DGR variable protein Mtd, which serves as the *Bordetella* bacteriophage’s receptor-binding protein. Sequence variation in Mtd enables *Bordetella* bacteriophage to keep pace with genetically programmed changes in its host *Bordetella*. A similar scenario is likely the case for ANMV-1 and its putative methanotrophic archaeal host. Mtd has a C-type lectin (CLec)-fold, and in particular belongs to the formylglycine-generating enzyme (FGE) subclass of the CLec-fold [14]. The CLec-fold is a general ligand-binding motif [15], but can also have enzymatic functionality as seen in FGE [16] and in sulfoxide synthase [17]. The only other structurally characterized DGR variable protein, TvpA from the spirochete *Treponema denticola*, also has an FGE-type CLec-fold [14]. Many bacterial and bacterial virus DGR variable proteins are predicted to have CLec-folds [18], while some others are predicted to have immunoglobulin (Ig)-folds [5, 7]. In contrast to these DGR variable proteins and the ANMV-1 variable protein, the archaeal DGR variable proteins [8] were unclassifiable based on sequence [19] or predicted structure [13].

We previously reported initial characterization of one of the archaeal DGR variable proteins, which we call here AvpA (Archaeal variable protein A; OTU1, Contig 3 DGR2) [8]. AvpA has only 15 and 8 % sequence identity to Mtd and TvpA, respectively, and its structure could not be predicted through *in silico* methods [13]. To determine how AvpA accommodates massive sequence variation, we determined its crystallographic structure. We find that AvpA has a CLec-fold, but one that is distinct from those of Mtd and TvpA. Capitalizing on the new structural information, we also identified AvpA-like proteins in metagenomes of marine and groundwater organisms. Significantly, most of the AvpA-like proteins from groundwater organisms belonged to putative DGRs. These results reveal that the CLec-fold is utilized to accommodate massive sequence variation widely, being conserved not only in viruses and bacteria but also in archaea.

## Results

### Overall structure

**Fig. 2:**
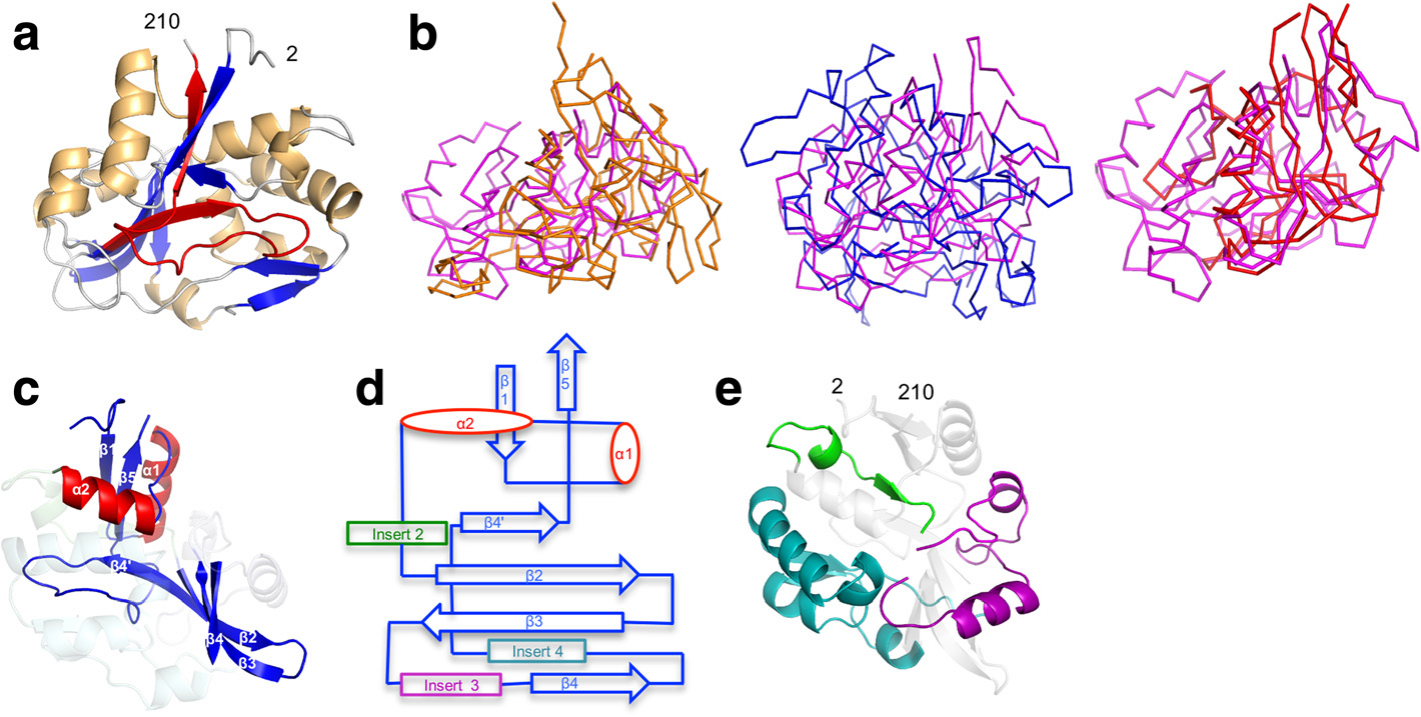
Structure of AvpA. **a** AvpA in ribbon representation (α-helices gold, β-strands blue, loops grey, and VR red). The amino acid positions of the N- and C-termini of the AvpA model are indicated. **b** Structural superposition of AvpA (*magenta*) with Mtd (*orange, left*), TvpA (*blue, middle*), and CLEC5A (*red, right*). **c** The core elements of the CLec-fold in AvpA in ribbon representation (α-helices red, β-strands blue). The inserts are ghosted. **d** Topology diagram of the CLec-fold in AvpA. **e** Inserts of AvpA in ribbon representation (insert 2, residues 41–57, green; insert 3, residues 89–114, purple; insert 4, residues 120–179, teal). The core elements of the CLec-fold are ghosted

AvpA was expressed in *Escherichia coli*, purified, and crystallized. The structure of AvpA was determined by single-wavelength anomalous dispersion (SAD) from selenomethionine-labeled AvpA and refined to 2.52 Å resolution limit (Table 1). The electron density calculated from SAD phases enabled residues 2–210 of AvpA to be traced, while electron density for residues 211–256 was absent, most likely due to the flexibility of this region. AvpA was a monomer in solution (data not shown) and in the crystal (Fig. 2).

**Table 1:** Data collection, phasing and refinement statistics for AvpA

	AvpA
Data collection	
Space group	P6_1_
Cell dimensions	
*a*, *b*, *c* (Å)	144 144 59.26
α, β, γ(°)	90, 90, 120
Wavelength	0.979 Å
Resolution (Å)	124.88–2.52(2.61–2.52)^a^
*R*_merge_	0.25(1.00)
*I* / σ_*I*_	12.5(1.8)
Completeness (%)	99.8(99.9)
Redundancy	7.4(6.9)
cc_1/2_	0.99(0.64)
Refinement	
Resolution (Å)	72.00–2.52 (2.55–2.52)
No. reflections	46142 (1638)
*R*_work_ / *R*_free_	0.20(0.34)/0.25(0.37)
No. atoms	
Protein	3422
Ligand/ion	4
Water	116
*B*-factors	
Protein	23.1
Ligand/ion	40.4
Water	42.5
R.m.s deviations	
Bond lengths (Å)	0.009
Bond angles (°)	1.26
MolProbity score	2.3[87^th^]^b^
Ramachandran	
% preferred	91.5
% allowed	7.5
% disallowed	1
Clashscore	11.7 [93^rd^]

^a^Highest resolution bin in parentheses here and other rows

^b^Percentile in brackets here and other rows

The structure of AvpA revealed a single globular domain that has a CLec-fold (Fig. 2). However, the CLec-fold in AvpA differed in detail from the FGE subclass of the CLec-fold seen in Mtd and TvpA. While the root-mean-square deviation (rmsd) in protein backbone among Mtd, TvpA, and human FGE was in the range of 1.9–2.6 Å [14], the rmsd between AvpA and Mtd was 3.4 Å (98 Cα; Z = 2.6), and between AvpA and TvpA 4.1 Å (92 Cα; Z = 2.5) (Fig. 2b). Likewise, AvpA was only distantly related to human FGE: rmsd of 4.0 Å (93 Cα; Z = 3.2). The strongest similarity of AvpA to a structurally characterized protein was to the mammalian protein CLEC5A (rmsd 2.8 Å, 98 Cα; Z = 5.6; 9 % sequence identity) (Fig. 2b). However, Mtd and TvpA also have similar levels of structural relationship to CLEC5A: rmsd of 2.9 Å for Mtd (90 Cα; Z = 5.6; 12 % sequence identity), and 2.6 Å for TvpA (92 Cα; Z = 5.5; 9 % sequence identity). Thus, while AvpA clearly has a CLec-fold, it is only distantly related to Mtd and TvpA, and likely represents a new subclass of the CLec-fold.

The CLec-fold in AvpA begins at residue 8 and continues to residue 209. The N- and C-terminal segments of this span form the characteristic CLec-fold pair of hydrogen-bonding, anti-parallel β-strands (β1 and β5) (Figs. 2c, d). In between these strands are other characteristic features of DGR CLec-fold proteins, such as two α-helices (α1 and α2) that are roughly perpendicular to each other, and a four-stranded, anti-parallel β-sheet (β2β3β4β4’), part of which forms the ligand-binding site [20]. Lastly, as in Mtd and TvpA, these secondary structure elements in AvpA are interrupted by inserts (Figs. 2d, e, and see below).

### Variable region

The variable region of AvpA (residues 181–203) is located close to but not at the very C-terminus of the protein, as it is for Mtd and TvpA. This internal location is common for the other identified *Nanoarchaeota* DGR variable proteins [8]. Forty-six amino acids follow the VR in AvpA. Electron density for this 46-residue extension, which is predicted by *in silico* methods to form two α-helices [13], was absent, most likely due to disorder or flexibility of this region. The DNA coding sequence for this 46-residue extension contains the putative hairpin/cruciform structure (Fig. 1), which in bacterial DGRs is typically located in the noncoding region following the VR. The hairpin/cruciform structure in AvpA is predicted by *in silico* methods [13] to encode five disordered amino acids [13], and thus its DNA sequence is unlikely to be constrained by the need to encode specific amino acids that are required for structural or functional reasons.

**Fig. 3:**
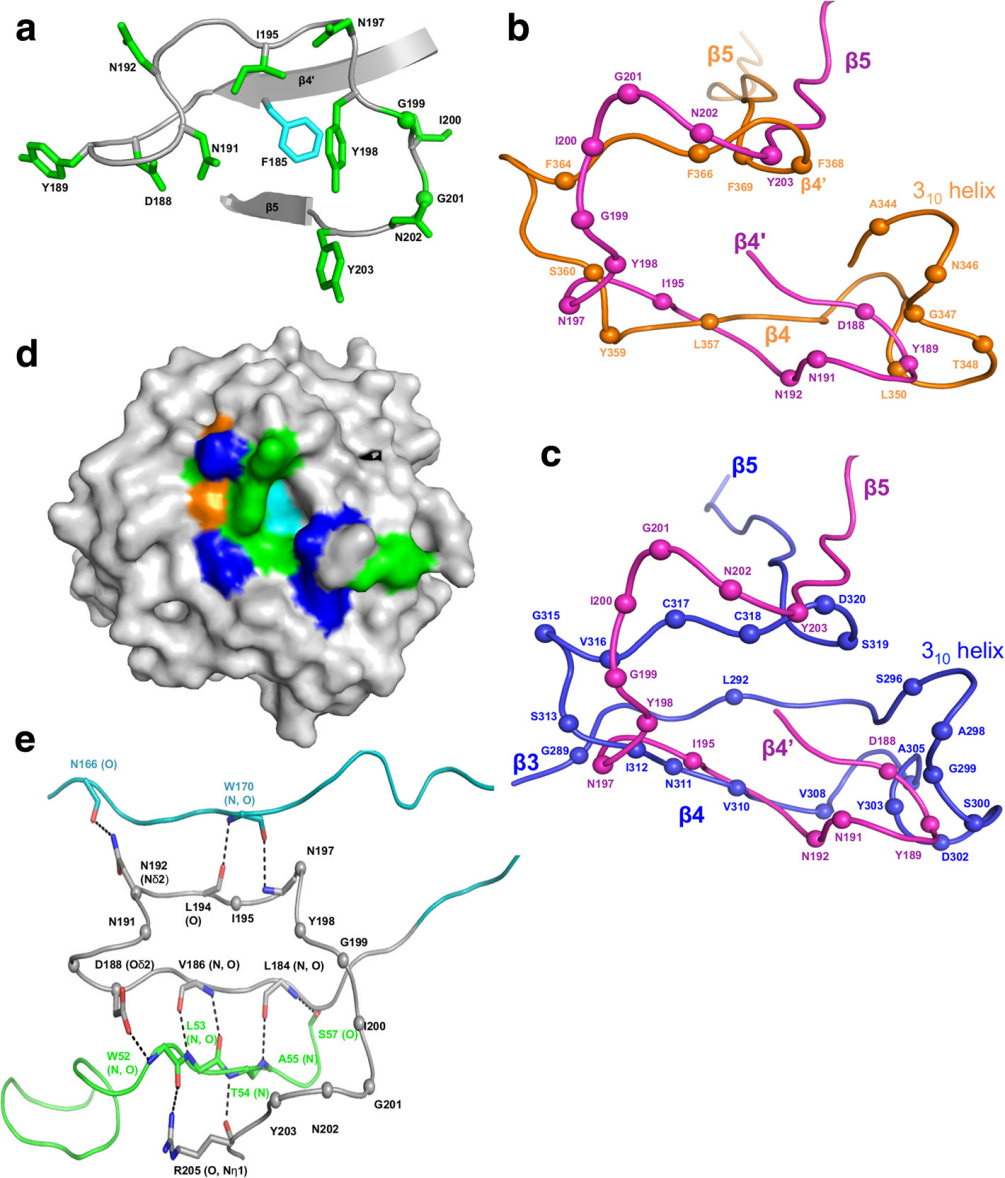
Variable Region. **a** Variable region of AvpA in ribbon representation. The main chain is gray and side chains of variable residues are green (green spheres correspond to glycines. The nonvariable residue F185 is shown in cyan. **b** Superposition of the VR of AvpA (*magenta*) and Mtd-P1 (*orange*) in Cα representation. The spheres represent variable amino acid positions. Secondary structure elements are labeled. **c** Superposition of the VR of AvpA (*magenta*) and TvpA (*blue*) in Cα representation. The representation is as in panel b. **d** Surface representation of AvpA, with variable hydrophobic residues (Y, I) green, variable hydrophilic residues (D, N) blue, variable glycines orange, and nonvariable F185 cyan. e Stabilization of the AvpA VR (*gray*) by insert 2 (*green*) and insert 4 (teal) in Cα representation. Hydrogen bonds are indicated with dashed lines

The variable regions of Mtd and TvpA were closely superimposable, despite their weak sequence identity of 16 % [14] (Table 2). In contrast, the variable region of AvpA differs substantially in conformation from those of Mtd and TvpA (Figs. 3a-c and Table 2). A major difference is that the variable residues of AvpA do not occur until the end of the β4’ strand, whereas variable residues are found as early as the β3 strand or just after the β3 strand for TvpA and Mtd, respectively. As expected from this difference, the 27 residue-length of the AvpA VR is about half that of Mtd and TvpA. Nevertheless, AvpA has 12 variable residues — the same number as in Mtd. These residues have the potential of generating 10^13^ variants, as 10 of the 12 have AAY codons, which as previously noted capture the gamut of chemistry and permit no stop codons [18]. These 12 variable residues were organized by the CLec-fold into a potential ligand-binding site (Fig. 3d), with a nonvariable aromatic amino acid (Phe 185) positioned centrally at the base of the binding site. A nonvariable aromatic amino acid also occurs centrally at the base of the ligand-binding sites in Mtd and TvpA, and in Mtd was seen to be involved in ligand binding [20]. This amino acid presumably provides a constant element of binding energy through hydrophobic contacts. The last portion of the VR is encoded by the nonvariant IMH element, which, as in Mtd and TvpA, encodes the nonvariant β5 strand.

**Table 2:** Comparison of the AvpA VR with equivalent regions of DGR and non-DGR proteins

		No. of equivalent residues	rmsd	*P*-value
AvpA	Mtd	29	2.72	0.26
AvpA	TvpA	9	1.51	0.58
AvpA	hFGE	30	3.08	0.43
AvpA	CLEC5A	27	2.2	0.08
Mtd	TvpA	38	1.2	5.1e^−05^

### Inserts

TvpA has three inserts within the core CLec-fold. We number the inserts with reference to Mtd and TvpA, and thus the first insert in AvpA is 2, found in the same topological location between α2 and β2 as in Mtd and TvpA. The equivalents of insert 1 and 1’ are missing in AvpA. Insert 2 is short (residues 41–57) and is composed of a 3_10_-helix and β-strand. AvpA has two inserts not seen in Mtd and TvpA: Insert 3 (residues 89–114) between β3 and β4, which is composed of loops and two α-helices; and insert 4 (residues 120–179) between β4 and β4’, which is composed of a more complicated arrangement of α-helices and two antiparallel β-strands. CLEC5A also has an equivalent of insert 4, but the CLEC5A and TvpA inserts are not structurally related. Indeed, the inserts in AvpA have no structural relationship to other known structures. As in Mtd and TvpA, the inserts serve in part to bolster the VR. In the case of AvpA, both inserts 2 and 4 make hydrogen bonds to the main chain of the VR, with the majority of contacts coming from insert 2 (Fig. 3e).

### Conservation of CLec-fold in DGRs of groundwater organisms

**Fig. 4:**
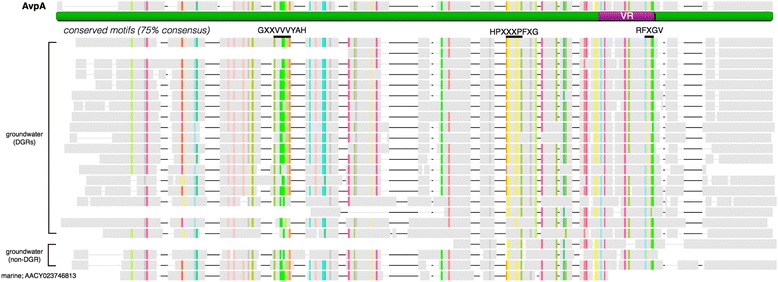
Conservation of CLec fold. Sequence alignment, including gaps, of AvpA and homologues from metagenomic studies. The sequence of AvpA is underlined in green and the variable region (VR) is shown in purple. Residues that are conserved in at least 75 % of sequences are highlighted; each amino acid is shown in a different color. The consensus sequence for selected conserved motifs is displayed below the AvpA sequence and highlighted with a solid black line

To determine whether proteins having similarity to AvpA exist in other genomes, a comprehensive search was conducted against public databases. Striking sequence conservation was observed between AvpA and representatives derived from both marine and groundwater metagenomes (Fig. 4). Our search revealed only a single homolog from marine metagenomes, but 22 non-redundant homologues from uncultivated, groundwater-associated organisms (Paul et al., in prep.). Among the groundwater matches, 19 sequences were derived from putative DGRs (Table 3), as they were proximal to a recognizable RT gene and a template region. Genes encoding the remaining three AvpA homologues do not appear to be parts of DGRs.

**Table 3:** List of homologous sequences that share pairwise sequence, and structural similarity to AvpA

		*AvpA Global Align*	*BackPhyre Analysis*		
	Name	Pairwise % id	Confidence (%)	Coverage (%)	
GROUNDWATER METAGNOME	id = 14867817	45	100	55	DGR-associated
	id = 522254	45	100	98	
	id = 14894049	43	100	97	
	id = 14867370	41	100	99	
	id = 14359504	41	100	96	
	id = 14904902	41	100	95	
	id = 15062502	40	100	96	
	id = 14403915	39	100	96	
	id = 14959041	39	100	97	
	id = 14859104	38	100	95	
	id = 14276933	37	100	96	
	id = 14881015	35	100	96	
	id = 14886209	31	100	92	
	id = 14985427	28	99.9	92	
	id = 14881050	23	97.7	94	
	id = 14875328	23	99.4	95	
	id = 12307670	22	97.7	33	
	id = 12129794	20	99.2	93	
	id = 14864751	17	94.7	77	
W.ARCH^a^	KHO51710	34	100	95	no DGR
	KHO51690	22	95.1	35	
	KHO52343	21	98.1	23	
	KHO52822	21	96.8	41	
	KHO52030	20	98.9	99	
	KHO46485	17	93.7	98	

^a^W.ARCH: Woesearchaeota genomes GW2011 AR3 and GW2011 AR17

We extended this inquiry by examining which sequences were likely to have folds related to that of AvpA using BackPhyre [13]. This analysis revealed additional sequences from groundwater metagenomes and archaeal genomes, which appear distantly related to AvpA (17 to 30 % pairwise similarity; Table 3). With this approach, AvpA relatives were identified in sequences of Archaeon GW2011 AR17 and Archaeon GW2011 AR3, both from uncultivated members of the phylum *Woesearchaeota* [21]. A sequence alignment with the least similar relative of AvpA revealed three conserved sequence motifs. The first was GXXVVVYAH (residues 67–75 in AvpA), which occupies the β3 strand with one side of the strand packing against insert 3 and the other side against insert 4. The second was HPXXXPFXG (residues 139–147 in AvpA), which resides in insert 4 as a short α-helix and packs against the β3 strand. The third was RFXGV (residues 205–209 in AvpA), which occupies the β5 strand and is encoded by the IMH element.

## Discussion

DGR variable proteins have evolved to accommodate massive sequence variation. This task is fulfilled in the adaptive immune system of jawed vertebrates by the Ig fold of antibodies and T cell receptors, and in the adaptive immune system of jawless vertebrates by the leucine-rich repeat fold of variable lymphocyte receptors. The first DGR variable protein to be structurally characterized was *Bordetella* bacteriophage Mtd. The crystal structure of Mtd revealed that its VR was organized into a ligand-binding site by a CLec-fold [18]. While sequence similarity among DGR variable proteins is strikingly low, an argument was made based on the structure of Mtd that several other DGR variable proteins were likely to have CLec-folds as well [18]. This prediction was confirmed by the crystal structure of one of these, *T. denticola* TvpA, which is capable of accommodating an astonishing 10^20^ sequences [14]. Although Mtd and TvpA share only ~16 % sequence identity, these proteins were both found to belong to the FGE subclass of the CLec-fold and have VRs that are remarkably similar in conformation. DGR variable proteins can apparently also adopt the Ig-fold [7], although direct structural verification of this prediction is not yet in hand. These putative Ig-fold proteins are predicted to have variable residues located on β-strand framework regions and in segments connecting Ig-fold domains, which is different from antibodies and T cell receptors, for which variable residues are sequestered to loops between β-strands.

A set of nine unique DGR variable proteins were identified in subterranean archaea related to *Nanoarchaeota* [8]. The sequence similarity among these proteins was low, and their folds were not predictable by *in silico* methods [13]. The results presented here on one of these, AvpA, revealed a remarkable conservation in archaea of the CLec-fold for accommodation of massive sequence variation. The AvpA CLec-fold was found to be divergent from those in Mtd and TvpA, with AvpA having a VR that differed considerably in conformation from the Mtd and TvpA VRs. These results are consistent with early divergence between bacterial and archaeal DGRs. AvpA-like proteins were also identified in metagenomes of uncultivated marine and groundwater organisms, with the majority of AvpA-like proteins in groundwater organisms belonging to putative DGRs. These groundwater metagenomes are rich in organisms representing archaeal phyla known to include ultra-small cells [9, 10, 21], raising the possibility that these DGRs also belong to nanosized organisms. In addition, AvpA-like proteins were identified in uncultivated members of *Woesearchaeota*, which have small genomes (~1000 protein coding genes) and limited metabolic capacities [21]. Thus, AvpA and AvpA-like proteins appear to occur in the DGRs of nanosized organisms, and while the function of AvpA and AvpA-like proteins is unknown, one likely possibility is to enhance symbiotic relationships between these minimal organisms and their hosts.

## Conclusions

These results have made apparent the widespread conservation of the CLec-fold in viruses, bacteria, and archaea for accommodating massive sequence variation. The fact that the CLec-fold in AvpA was not predictable by *in silico* methods points to the remarkable sequence space available to this fold. The great proportion of CLec-fold proteins occurs in metazoans, but this fold has also been observed in some viral and bacterial proteins other than DGR variable proteins [15]. To our knowledge, this is the first report of a CLec-fold protein occurring in archaea. The structure of AvpA did not provide further illumination on the protein folds of the other eight identified archaeal DGR variable proteins [8]. This indicates that there may yet be other folds by which DGR variable proteins accommodate massive sequence variation, or more likely given the resilience of the CLec-fold to primary sequence variation, these proteins may represent further cases of the CLec-fold occurring in archaea.

## Methods

### Crystallization and structure determination

Selenomethionine (SeMet)-substituted AvpA was expressed and purified as described [8], except *Escherichia coli* was cultured in synthetic minimal media supplemented with 200 mg/L L(+)-Selenomethionine (Sigma) [22]. Crystals of SeMet-labeled AvpA were grown by the hanging drop method at 20 ^o^C by mixing 1 μL of AvpA (50 mg/mL) and 1 μL of 30 % (v/v) PEG monomethyl ether 550, 50 mM MgCl_2_, 100 mM HEPES, pH 7.5. Crystals were cryoprotected by soaking in the precipitant solution supplemented with 10 % glycerol and 2 mM TCEP. Single-wavelength anomalous dispersion (SAD) data were collected at Advanced Photon Source (Argonne, IL) beamline 24-ID-E. Diffraction data were indexed, integrated, and scaled with MOSFLM [23–25]. Se sites were located from SAD data of SeMet-labeled AvpA, and initial phases were determined using SOLVE [26]. Out of the four methionines (M1, M16, M32 and M98), all but the first were located. The asymmetric unit was found to contain two molecules of AvpA.

A partial model of AvpA (residues 10–28, 45–120, 146–157, 178–187 and 193–202) was built by automatic means using Autobuild (within Phenix) into SAD phased electron density. Further model building was carried out manually with COOT [27], as guided by σ_A_-weighted 2mF_o_-DF_c_ and mF_o_-DF_c_ difference maps. A total of sixty-three iterative rounds of manual model building and maximum likelihood refinement were carried out with Refine (within Phenix) using default parameters [28, 29], with each refinement step consisting of 3–5 cycles. One round of TLS parameterization with default settings was then used, followed by the addition of water and magnesium ions into ≥3σ mF_o_-DF_c_ density. Structure validation was carried out with Molprobity [30], and molecular figures were generated with PyMOL (http://www.pymol.org/).

### Structural alignment of VR and equivalent regions

The structure of the VR of AvpA (residues 181–210) was compared to that of the VR of Mtd (residues 337–381) and TvpA (residues 285–329) using FATCAT [31]. For hFGE and CLEC5A, residues 322–369 and residues 158–187, respectively, were used for comparison. These regions of hFGE and CLEC5A are spatially equivalent to the VRs of the DGR variable proteins.

### Homologue search and DGR analysis

The amino acid sequence of AvpA was compared with representatives from public databases, including NCBI nr, env, and UniprotKB, using blastp, tblastn [19], and pHMMER [32], respectively. Multiple sequence alignment of homologues and AvpA was performed using ClustalW [33] and conserved motifs were visualized using Geneious v8.1 (Biomatters Ltd). Analysis of structural homology was performed using BackPhyre [13] with the structure of AvpA as a query, and proteins from nanoarchaeal genomes, *Woesearchaeota* genomes, and previously identified groundwater metagenome DGRs (Paul et al., in preparation).

Putative DGR sequences were detected in three steps. First, RT-containing sequences were identified using blastp versus known DGRs and relatives with an e-value cutoff of 1×10^−10^. Next, using a custom python script, near repeats were identified within 10 kb of the putative RT gene (i.e., VR and TR) with at least five adenine-specific mismatches and no more than one non-adenine mismatch. This step involved fragmenting the ~10 kb (+ RT) sequences using a sliding window of 200 bp and an overlapping step of 50 bp. Fragments were compared using blastall and near-identical repeats, whose mismatches exclusively consisted five or more adenine-variable sites, were recorded as putative VR/TR pairs.

## Abbreviations

Avd: Accessory variability determinant
AvpA: Archaeal variable protein A
Bb: *Bordetella* bacteriophage
CLec: C-type lectin
DGR: Diversity-generating retroelement
FGE: Formylglycine-generating enzyme
Ig: Immunoglobulin
IMH: Initiation of mutagenic homing
rmsd: Root-mean-square deviation
RT: Reverse transcriptase
SAD: Single-wavelength anomalous dispersion
SeMet: Seleno-methionine
TR: Template region
VR: Variable region
